# The Complexity and Diversity of the Pathogenicity Locus in *Clostridium difficile* Clade 5

**DOI:** 10.1093/gbe/evu248

**Published:** 2014-12-10

**Authors:** Briony Elliott, Kate E. Dingle, Xavier Didelot, Derrick W. Crook, Thomas V. Riley

**Affiliations:** ^1^Microbiology and Immunology, School of Pathology and Laboratory Medicine, The University of Western Australia, Crawley, Western Australia, Australia; ^2^Nuffield Department of Clinical Medicine, John Radcliffe Hospital, Oxford University, United Kingdom; ^3^National Institute for Health Research, Oxford Biomedical Research Centre, John Radcliffe Hospital, Oxford, United Kingdom; ^4^Department of Infectious Disease Epidemiology, Imperial College, London, United Kingdom; ^5^Division of Microbiology and Infectious Diseases, PathWest Laboratory Medicine, Nedlands, Western Australia, Australia

**Keywords:** evolution, phylogeny, toxin locus

## Abstract

The symptoms of *Clostridium difficile* infection are caused by two closely related toxins, TcdA and TcdB, which are encoded by the 19.6 kb Pathogenicity Locus (PaLoc). The PaLoc is variably present among strains, and in this respect it resembles a mobile genetic element. The *C. difficile* population structure consists mainly of five phylogenetic clades designated 1–5. Certain genotypes of clade 5 are associated with recently emergent highly pathogenic strains causing human disease and animal infections. The aim of this study was to explore the evolutionary history of the PaLoc in *C. difficile* clade 5. Phylogenetic analyses and annotation of clade 5 PaLoc variants and adjoining genomic regions were undertaken using a representative collection of toxigenic and nontoxigenic strains. Comparison of the core genome and PaLoc phylogenies obtained for clade 5 and representatives of the other clades identified two distinct PaLoc acquisition events, one involving a toxin A^+^B^+^ PaLoc variant and the other an A^−^B^+^ variant. Although the exact mechanism of each PaLoc acquisition is unclear, evidence of possible homologous recombination with other clades and between clade 5 lineages was found within the PaLoc and adjacent regions. The generation of nontoxigenic variants by PaLoc loss via homologous recombination with PaLoc-negative members of other clades was suggested by analysis of *cdu2*, although none is likely to have occurred recently. A variant of the putative holin gene present in the clade 5 A^−^B^+^ PaLoc was likely acquired via allelic exchange with an unknown element. Fine-scale phylogenetic analysis of *C. difficile* clade 5 revealed the extent of its genetic diversity, consistent with ancient evolutionary origins and a complex evolutionary history for the PaLoc.

## Introduction

The gram-positive, spore-forming bacterium *Clostridium difficile* is a major cause of healthcare-associated diarrhea, and the most commonly reported nosocomial pathogen in the United States (Magill et al. 2014). *Clostridium difficile* infection (CDI) encompasses a wide clinical spectrum ranging from asymptomatic carriage to mild self-limiting diarrhea, and ultimately fulminant colitis and toxic megacolon. The *C. difficile* population structure consists of six distinct phylogenetic clades designated 1, 2, 3, 4, 5, and C-I (Dingle et al. 2011, 2014; Stabler et al. 2012). Toxin production varies by clade; the most divergent clade, C-I, produces neither binary toxin, nor the two main toxins, toxin A and toxin B. Strains belonging to clades 2, 3, and 5 produce binary toxin, whereas most genotypes within clades 1–3 produce both toxins A and B. Clade 4 is largely nontoxigenic except for multilocus sequence type (MLST) ST37 (polymerase chain reaction [PCR]-ribotype 017) toxinotype VIII strains which only produce toxin B.

Clade 5 is noteworthy because it contains the clinically important PCR-ribotype 078 or ST11. This genotype causes illness which is more severe and associated with higher mortality rates than other clinically important strains (Walker et al. 2013). Clade 5 is also notable for its divergence from the first four clades, separating an estimated 85 Ma (He et al. 2010). Ribotype 078 has a strong association with animal infections (Rodriguez-Palacios et al. 2007; Pirs et al. 2008; Rupnik et al. 2008; Pelaez et al. 2013), and its incidence in cases of symptomatic human infection is increasing (Goorhuis et al. 2008; Bauer et al. 2011; Eckert et al. 2013). Less abundant clade 5 genotypes have also been identified in animals, including ribotypes 033 (toxinotype XIa/b), 045 (toxinotype XIV), 126 (toxinotype XXVIII), and 127 and 237 (toxinotype XXXI) (Schneeberg et al. 2012, 2013; Squire et al. 2013; Knight and Riley 2013; Knight et al. 2013).

The symptoms of CDI are caused by two closely related toxins, designated A and B, and encoded by the pathogenicity locus (PaLoc), a 19 kb chromosomally integrated DNA sequence (Braun et al. 1996). The PaLoc’s single genomic location (Braun et al. 1996; Dingle et al. 2011), clade-specificity of its genetic variants (Dingle et al. 2011), and a lack of genes conferring mobility (Braun and von Eichel-Streiber 1999) suggest that it is stably integrated. However, nontoxigenic strains exist which intermingle phylogenetically with toxigenic strains of clades 1, 4, and 5, suggesting multiple gain and loss events (Elliott et al. 2009; Dingle et al. 2011, 2014). Transfer of the PaLoc to nontoxigenic strains and resultant toxigenic conversion has been demonstrated experimentally (Brouwer et al. 2013).

The *C. difficile* toxins belong to the large clostridial toxin family, members of which have also been identified in *Clostridium sordellii*, *Clostridium novyi*, and *Clostridium perfringens* (Amimoto et al. 2007). The large clostridial toxins comprise four domains (Davies et al. 2011): A glucosylating enzymatic domain, an autocatalytic processing/protease domain, a membrane-translocating domain and a repetitive, and receptor-binding domain. Because of the large size of the toxin-encoding genes, analysis of genetic polymorphism within the *C. difficile* PaLoc has frequently been confined to toxinotyping, a method based on PCR and restriction endonuclease digests which infers nucleotide sequence variation indirectly (Rupnik et al. 1998). Within clade 5, toxinotypes are largely congruent with genotype (Stabler et al. 2012); for example, the 078 PaLoc is designated toxinotype V. Deletions which can abrogate toxin production (Rupnik 2008) are common within the repetitive sequences of the toxin A receptor-binding domain and have been described in clades 1, 2, 4, and 5 (Rupnik 2008; Zaiß et al. 2009). The PaLoc encodes a further three genes: *tcdR* (an alternative sigma factor), *tcdE* (a bacteriophage-like putative holin), and *tcdC* (a negative regulator), and contains a phage-like endolysin fragment (Monot et al. 2011). Changes to PaLoc genes, most notably *tcdB* (Lanis et al. 2013) and more controversially, *tcdC* (Carter et al. 2011; Bakker et al. 2012; Cartman et al. 2012), have been proposed to impact on the severity of clinical disease.

The purpose of this study was to explore the evolutionary history of the PaLoc in *C. difficile* clade 5. To do so, the phylogeny of clade 5 was reconstructed based on whole-genome sequences (WGS) from a representative collection of isolates comprising both toxigenic and nontoxigenic strains. The toxigenic strains within clade 5 exhibited significant PaLoc variation consistent with the previously assigned toxinotypes XIa/b, XIV, XXVIII, and XXXI (Schneeberg et al. 2012, 2013; Squire et al. 2013; Knight and Riley 2013; Knight et al. 2013). The extent of clade 5 PaLoc variation was studied and compared with this locus in members of other clades. By examining the whole genome and PaLoc phylogenies, we aimed to understand the evolutionary events leading to PaLoc acquisition and/or loss by clade 5.

## Materials and Methods

### Isolates and Genome Sequencing

A total of 23 isolates from clade 5 were included in the study. The reference isolate L033 was kindly supplied by Prof. Mark Wilcox, Leeds Institute of Molecular Medicine, University of Leeds, United Kingdom cultured originally from a patient in the Netherlands. The WGS of this isolate was reported previously (Dingle et al. 2013). All other isolates were from Australia and included 16 human clinical isolates from: Western Australia (*n* = 9); New South Wales (*n* = 1); Victoria (*n* = 1); and Queensland (*n* = 1). These were isolated between December 11, 2005 and August 7, 2010, except for HCD52 which was first cultured in 1998. The Western Australian human isolates were cultured at the state pathology laboratory, PathWest, while the others were referred to PathWest from diagnostic laboratories in other states for PCR ribotyping. Animal isolates (*n* = 6) of bovine (*n* = 1), equine (*n* = 1), and porcine (*n* = 4) origin, cultured between March 26, 2007 and November 23, 2009 in New South Wales and Western Australia, were also included. The presence of PaLoc sequences within the isolates was determined previously using published PCR assays for *tcdA* (including both the repetitive and nonrepetitive regions; Kato et al. 1998), *tcdB* (Kato et al. 1991), and for both binary toxin component genes *cdtA* and *cdtB* (Stubbs et al. 2000).

Genomes were sequenced using Illumina Technology (Bentley et al. 2008) as described previously (Dingle et al. 2013). De novo assemblies were constructed using Velvet (Zerbino and Birney 2008) and A5 (Tritt et al. 2012). All the isolates had previously been toxin genotyped by PCR to detect the presence of the toxin A and toxin B genes, *tcdA* and *tcdB*, respectively, with regions in both the repetitive and nonrepetitive domain of *tcdA* targeted (Kato et al. 1991, 1998). The presence of both binary toxin genes was also tested for by PCR (Stubbs et al. 2000). Previously sequenced genomes for strain CD630 (UK 012, ST54, A^+^B^+^CDT^−^) (Sebaihia et al. 2006), Ox160 (UK 027, ST1, A^+^B^+^CDT^+^), Ox1485 (UK 023, ST5, A^+^B^+^CDT^+^), and Ox920 (UK 017, ST37, A^−^B^+^CDT^−^) were used as references for clades 1–4, respectively (Dingle et al. 2014).

### Phylogenetic Analysis

All isolates were PCR ribotyped at PathWest using published oligonucleotide primers (O'Neill et al. 1996), and the products were separated by capillary electrophoresis and patterns compared against the local reference library. A subset (*n* = 8) that did not match any of the previously determined banding patterns available in Australia was referred to the United Kingdom Anaerobe Reference Unit (University Hospital of Wales, Heath Park, Cardiff, Wales, UK) for comparison against their library. Seven were found to be novel types and assigned new UK PCR-ribotype designations. Following the move of the UK *C. difficile* reference library to the Microbiology Department, Leeds General Infirmary (Leeds, England, UK), a further eight isolates were referred for typing, six of which were novel and assigned new UK designations. A subset of isolates chosen to represent closely phylogenetically related clade 5 groups on the basis of their PCR-ribotyping patterns, underwent toxinotyping using the A3 and B1 fragments, as previously described (Rupnik et al. 1998). Data were extracted bioinformatically from unclosed WGS using BIGSdb (Jolley and Maiden 2010) to determine MLST as per a published scheme (Griffiths et al. 2010). The sequence type (ST) was determined for each isolate using the database available at http://pubmlst.org/cdifficile. Newly identified alleles and STs were submitted to the database and assigned numbers in order of discovery. Fine-scale phylogenies were constructed using the core genomes for all clade 5 isolates. These were constructed using ClonalFrame (Didelot and Falush 2007), and ancestry times were calculated in years using isolate dates and previous estimate of the *C. difficile* molecular clock (Didelot et al. 2012). Polymorphisms between four pairs of isolates were mapped along the whole M120 genome (Sebaihia et al. 2006) by aligning their de novo assemblies against M120 using MuMMER version 3.25 (Kurtz et al. 2004). The distribution of polymorphisms in the PaLoc region was plotted using DNAplotter (Carver et al. 2009).

### Gene Prediction, Annotation, and Comparison

The PaLoc region and insertion sites were studied in detail, sequences being translated and annotated using Artemis genome browser and annotation tool (Rutherford et al. 2000). Sequence comparisons were performed using the Artemis Comparison Tool (Carver et al. 2005). Analysis of the PaLoc genes *tcdR*, *tcdB*, *tcdA, tcdC*, and *tcdE* was performed using MEGA 5.2 (Tamura et al. 2011) to construct neighbor-joining trees. BLAST searches of genes and predicted translation products against GenBank were used to identity their putative functions. Secondary structure prediction of nucleic acids was performed using the RNAfold server (Gruber et al. 2008), and putative RNA genes searched against the Rfam database (Griffiths-Jones et al. 2003).

## Results

### Isolates and Genotypes

*Clostridium difficile* isolates identified during previous studies as belonging to clade 5 on the basis of MLST data or PCR ribotype were chosen (*n* = 23) (Elliott et al. 2009; Elliott et al. 2011; Stabler et al. 2012; Squire et al. 2013; Elliott B, Hong S, Knight DR, Riley TV, unpublished data). The presence of the PaLoc and therefore the genetic potential for toxin expression had been assessed previously by PCR assay for the presence of toxin genes. Isolates which were PCR positive for *tcdA* and/or *tcdB* are subsequently referred to as toxigenic and designated A^+^B^+^ or A^−^B^+^. Isolates for which toxin gene PCR assays were negative, and the lok1/3 PCR (Braun et al. 1996) was positive were designated either nontoxigenic or A^−^B^−^. The absence of toxin A production was confirmed by enzyme immunoassay for isolates in which *tcdA* deletions were detected, with the Triage *C. difficile* Panel (Biosite). Production of toxin B was confirmed using the cell culture cytotoxicity assay as previously described (Bowman and Riley 1986). All isolates (toxigenic [*n* = 18] and nontoxigenic [*n* = 5]) were genotyped by MLST; a total of nine STs were identified, eight of which were novel (table 1). No ST occurred in both toxigenic and nontoxigenic variants. Corresponding PCR ribotype and toxinotyping data are indicated in table 1. Four STs were represented by only one isolate and one ST by two isolates belonging to the same ribotype. The other four STs contained both multiple isolates and PCR ribotypes (table 1). ST11 was unusual in being further discriminated into four PCR-ribotypes.

**Table 1:** Isolate Details

Isolate Code	Isolate No.	Collection Date	Host	State	Toxin Profile	Ribotype	Toxinotype	ST
ES 153	06-4555666	July 19, 006	Human	NSW	A ^+^ B ^+^ CDT^+^	UK 126	ND	11
ES 210	JH09M67811	July 29, 2009	Human	NSW	A ^+^ B ^+^ CDT^+^	UK 126	ND	11
AI 155	H.WA2	August 2009	Equine	NSW	A ^−^ B ^+^ CDT^+^	UK 605	ND	163
ES 223	JH10M3970	January 12, 2010	Human	NSW	A ^−^ B ^+^ CDT^+^	UK 606	ND	163
WA 16	P06-4500199M	January 5, 2006	Human	WA	A ^−^ B ^+^ CDT^+^	UK 584	ND	163
HCD 52	Cdiff 642	1998	Human	WA	A ^−^ B ^−^ CDT^+^	UK 585	NT	164
Q 6	324060196	May 3, 2007	Human	QLD	A ^−^ B ^−^ CDT^+^	UK 586	NT	167
AI 6	07-321	March 26, 2007	Porcine	WA	A ^−^ B ^−^ CDT^+^	UK 238	NT	169
WA 12	Q05-3910805C	December 11, 2005	Human	WA	A ^−^ B ^−^ CDT^+^	UK 239	NT	168
WA 94	P06-9511410S	January 29, 2006	Human	WA	A ^+^ B ^+^ CDT^+^	UK 078	V	11
ES 280	98700735	August 7, 2010	Human	VIC	A ^+^ B ^+^ CDT^+^	UK 078	V	11
RPH 123	P07-9572883U	June 28, 2007	Human	WA	A ^+^ B ^+^ CDT^+^	UK 078	V	11
WA 27	P06-1321417R	January 31, 2006	Human	WA	A ^+^ B ^+^ CDT^+^	UK 127	VI	11
L033		Unknown	Human	UK	A ^−^ B ^−^ CDT^+^	UK 033	XI	11
WA 13	Q05-3033322T	December 31, 2005	Human	WA	A ^−^ B ^+^ CDT^+^	UK 291	XXX	164
ES 30	06-081-2908	March 22, 2006	Human	NSW	A ^−^ B ^+^ CDT^+^	UK 280	XXX	166
PMH 25	P07-7325065W	August 19, 2007	Human	WA	A ^+^ B ^+^ CDT^+^	UK 280	XXX	166
AI 27	0048	October 9, 2008	Bovine	NSW	A ^−^ B ^+^ CDT^+^	UK 583	XXX	173
ES 166	06-5509002	September 20, 2006	Human	NSW	A ^−^ B ^+^ CDT^+^	UK 281	XXX	174
AI 15	P06-2941A	April 18, 2007	Porcine	WA	A ^−^ B ^+^ CDT^+^	UK 237	XXXI	167
AI 152	R11-GG1	November 23, 2009	Porcine	WA	A ^−^ B ^+^ CDT^+^	UK 285	XXXI	167
AI 178	GG1-FS(1)	October 15, 2009	Porcine	WA	A ^−^ B ^+^ CDT^+^	UK 237	XXXI	167
WA 151	P06-4574276A	July 11, 2006	Human	WA	A ^−^ B ^+^ CDT^+^	UK 237	XXXI	167

Note.— ND, not done; NT, nontoxigenic (i.e., PaLoc-negative); NSW, New South Wales; WA, Western Australia; QLD, Queensland; VIC, Victoria; UK, United Kingdom.

### Genetic Organization of the Clade 5 PaLocs

Unclosed WGS were determined for the 23 isolates. The closed genomes of clade 5 strain M120 (ST11) (He et al. 2010) and clade 1 reference strain 630 (ST54) (Sebaihia et al. 2006; Monot et al. 2011) were used for comparative purposes (GenBank accession numbers FN665653 and AM180355, respectively). They provided scaffolds against which the de novo assembled clade 5 PaLoc sequences could be aligned for the toxigenic strains. The unclosed genome sequence of isolates Ox160, Ox1485, and Ox920 were used as references for clades 2, 3, and 4, respectively (Dingle et al. 2011, 2014).

The region of the chromosome containing the PaLoc was annotated in toxigenic strains and the PaLoc insertion site in nontoxigenic strains. All 18 isolates previously designated toxigenic by PCR contained PaLoc sequences of 10,211–19,742 kb located within the single chromosomal integration site described previously (Braun et al. 1996). Of these, 12 isolates were negative for *tcdA* and designated A^−^B^+^, whereas the remainder was A^+^B^+^. These data suggested the possibility that clade 5 contained two phylogenetically distinct PaLoc variants.

**Fig. 1.—:**
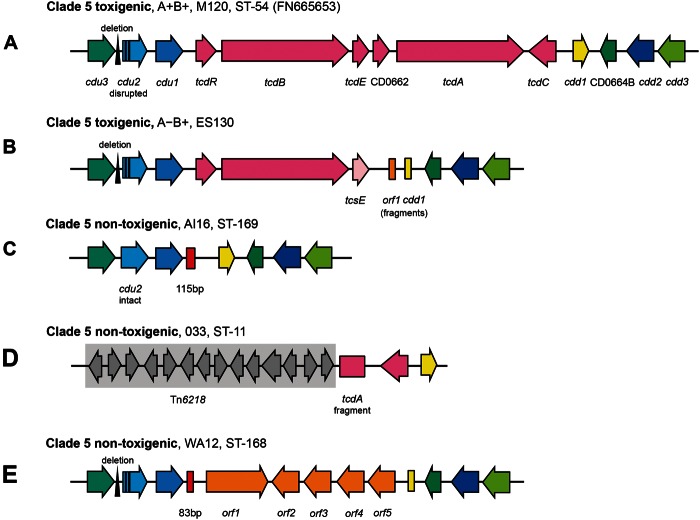
Distinct variants of the PaLoc found in clade 5. The genetic organization of the clade 5 PaLoc and flanking genes: (*A*) A^+^B^+^, toxinotypes XXX and XXXI, (*B*) A^−^B^+^, (*C*) PaLoc-negative strains, (*D*) A^−^B^−^ toxinotype XI strains, and (*E*) WA12 containing nontoxigenic clade C-1 related (non-PaLoc) sequence at the PaLoc integration site and consequently A^−^B^−^.

The genetic organization of the PaLoc in clade 5 was investigated. Within the six clade 5 A^+^B^+^ isolates the five PaLoc open-reading frames (ORFs) were intact, resembling typical PaLocs as exemplified by the reference genomes used comparatively (fig. 1*A*; Sebaihia et al. 2006). Sequence analysis of the A^−^B^+^ clade 5 isolates (toxinotypes XXX and XXXI) identified their common genetic organization and a large deletion within the PaLoc (10,366 bp relative to reference M120) encompassing *tcdA* and *tcdC* (fig. 1*B*).

### The PaLoc Integration Site in Nontoxigenic Clade 5

Investigation of the empty clade 5 PaLoc integration site in the five nontoxigenic isolates revealed that the majority (3/5) lacked the entire PaLoc, containing the 115 bp sequence described previously in its place (Braun et al. 1996; fig. 1*C*), among nontoxigenic isolates of clades 1 and 4 (Dingle et al. 2014). The first exception was an ST11(033) isolate that contained a PaLoc fragment consistent with toxinotype XI (Geric and Rupnik 2010; fig. 1*D*): only *tcdC* and the 3′-terminal 2,456 bp of *tcdA* containing a 681 bp deletion in the repetitive sequences were present, together representing 6,521 bp of the PaLoc. This reflected a 51,379 bp deletion relative to strain M120 (He et al. 2010), removing *tcdR*, *tcdB*, *tcdE*, and part of *tcdA* as well as the region immediately upstream of the PaLoc. A variant of the mobile element Tn*6218* (Dingle et al. 2014) was present immediately upstream of this element in ST11(033) (fig. 1*D*). The second exception was the previously described isolate WA12 (fig. 1*E*) containing a 7,183 kb sequence at the PaLoc integration site (Elliott et al. 2009). A closely related sequence (87% nt sequence identity) is also present in nontoxigenic clade C-I strains (Dingle et al. 2014).

### Inter and Intraclade Phylogenetic Relationships of the Clade 5 PaLoc

**Fig. 2.—:**
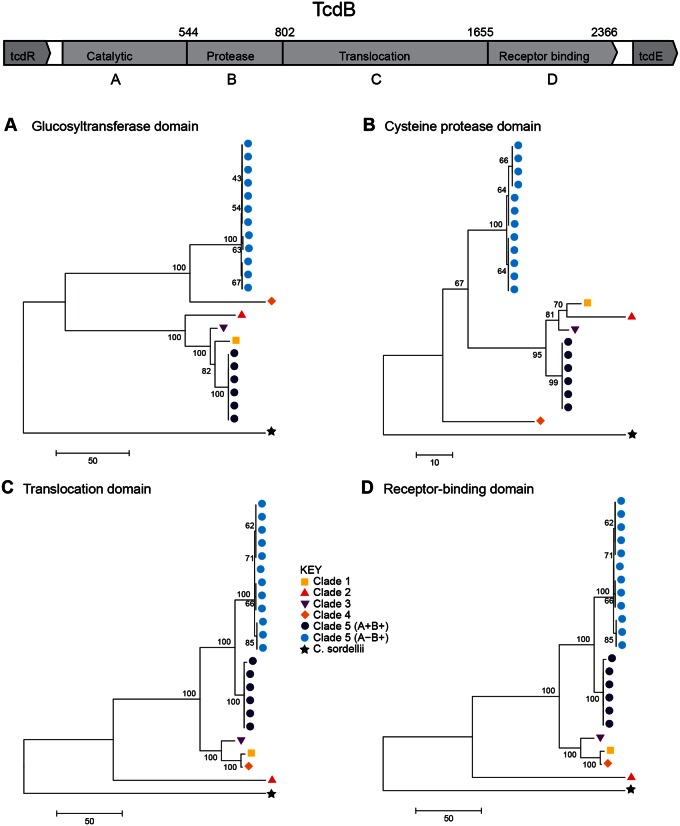
Independent clade 5 PaLoc acquisitions are supported by the crosspopulation phylogeny of *tcdB* functional domains. Phylogenies constructed from the four domains of *tcdB*: (*A*) The glucosyltransferase domain, (*B*) the cysteine protease domain, (*C*) the translocation domain, and (*D*) the receptor-binding domain. Colored labels indicate clade (see Key). Strain labels are shown in supplementary figure S1, Supplementary Material online.

**Fig. 3.—:**
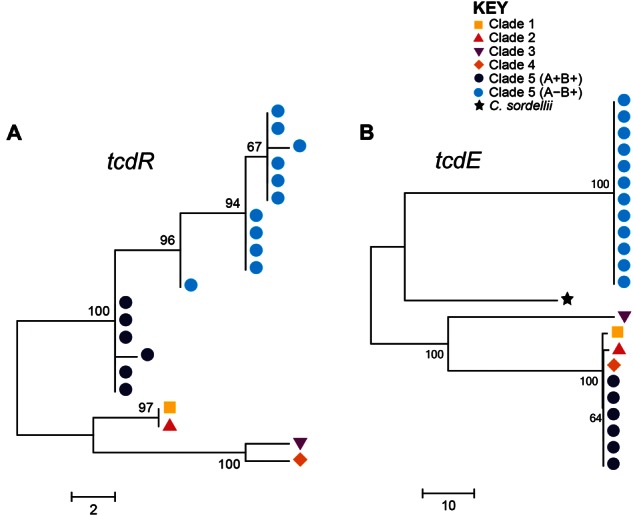
Phylogenetic relationship of PaLoc accessory genes. Phylogenies of the PaLoc accessory genes (*A*) *tcdR* and (*B*) *tcdE*, which encode a positive regulator and a putative holin gene, respectively. The A^−^B^+^ *tcdR* gene (from strain AI27, marked with asterisk) clustered closest to the A^+^B^+^ lineage (*A*) was a hybrid of the two sequences suggesting it derived from a recombination event (data not shown). The putative holin gene present in the PaLoc of clade 5 A^−^B^+^ strains is distantly related to that found in the other clades (*B*), suggesting it was gained via allelic exchange with another source. Strain labels are shown in supplementary figure S2, Supplementary Material online.

The *tcdB* gene was present and intact in all 18 toxigenic clade 5 PaLoc variants, and was therefore used for focused investigation of the PaLoc phylogenetic relationships with one another and with representatives from other clades (fig. 2*A–D*). This analysis identified two distinct clade 5 PaLoc variants corresponding to A^+^B^+^ and A^−^B^+^, each of which was genetically homogeneous. The genetic distance between the two clade 5 variants changed across *tcdB* (fig. 2*A–D*). They were clearly distinct from one another within the glucosyltransferase and protease domains (fig. 2*A–B*), but more closely related within the translocation and receptor-binding domains (fig. 2*C–D*). The only other intact gene to be present in all toxigenic clade 5 PaLoc variants, *tcdR*, also supported this separation between A^+^B^+^ and A^−^B^+^ (fig. 3*A*). The *tcdR* gene of isolate AI27, which was intermediate between the two groups, was a hybrid of the two. The glucosyltransferase domain of the A^−^B^+^ clade 5 strains was notable for its homology to clade 4 ST37(017), which is also A^−^B^+^ (fig. 2*A*). These data suggest an evolutionary history involving recombination of PaLoc sequences involving clade 5 and members of other clades.

### Clade 5 PaLoc Acquisition

**Fig. 4.—:**
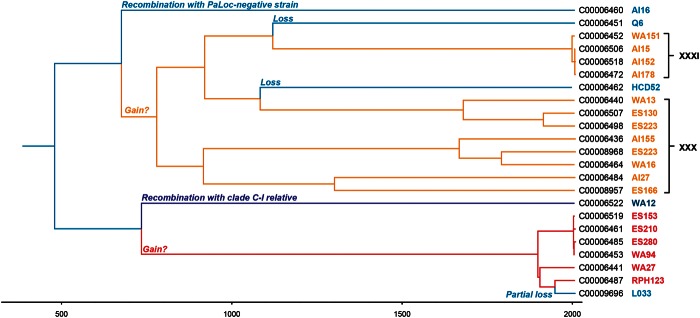
The two PaLoc variants were each acquired independently approximately 1,300 years ago. A time-scaled ClonalFrame tree of the core genome dating (approximately) the events of acquisition and loss of the PaLoc within clade 5. The different lineages are color coded according to toxin profiles: A^+^B^+^ (red), A^−^B^+^ (orange), and A^−^B^−^ (blue). The A^−^B^+^ isolates within clade 5 can be divided into two separate branches based on toxinotype (shown in black text on the right).

A ClonalFrame tree was constructed using the core genomes of all clade 5 isolates (fig. 4). This tree was time scaled to allow the possibility of approximately dating the evolutionary events which led to the current distribution of the PaLoc within clade 5. The most parsimonious scenario involves separate gains of the A^−^B^+^ and A^+^B^+^ PaLoc variants and several subsequent losses. The acquisition of the A^−^B^+^ variant was dated at approximately 1,300 years ago whereas the gain of the A^+^B^+^ variant could either be ancient, or as recent as approximately 100 years ago due to its occurrence on a very long branch.

**Fig. 5.—:**
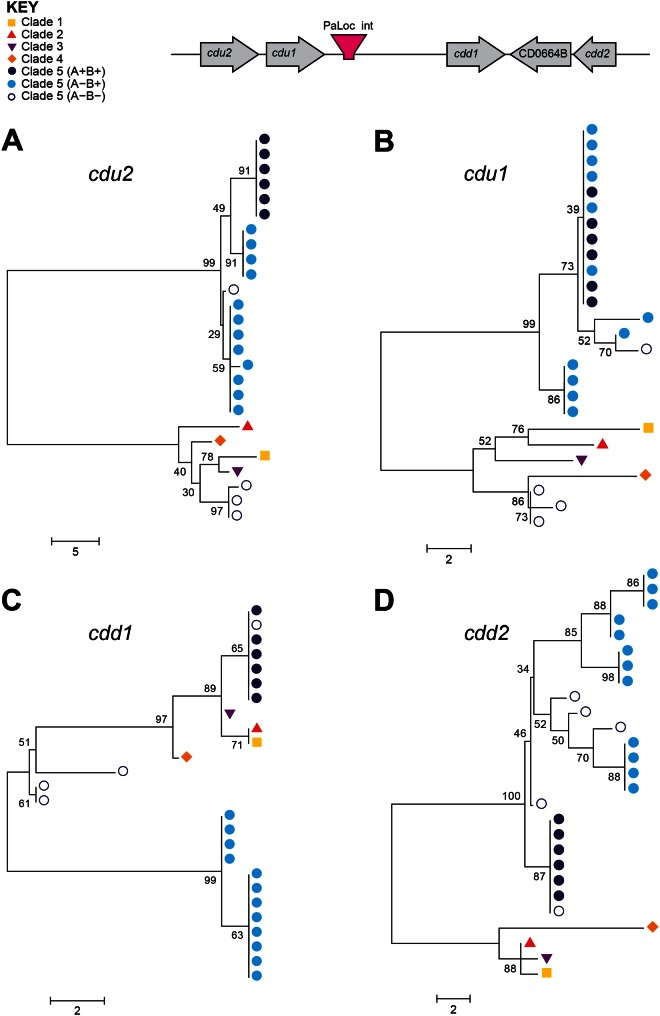
Acquisition and loss of the clade 5 PaLoc via homologous recombination. In A^−^B^−^ clade 5 strains, the phylogenies of *cdu2* (*A*) and *cdu1* (*B*) genes upstream of the PaLoc are distinct from other clade 5 strains, with the exception of WA12. The genes seen in these strains are more closely related to those of clades 1–4, suggesting that the PaLoc was lost via homologous recombination with a nontoxigenic strain from one of these clades. In the A^+^B^+^ clade 5 strains, the same is seen with the *cdd1* gene *(C)*, suggesting these strains gained *cdd1* along with the PaLoc via homologous recombination with a toxigenic strain from another clade, whereas the phylogeny of *cdd2 (D)* remains relatively consistent throughout clade 5. L033 is not included in the trees for *cdu2* and *cdu1* as these genes are not present in this strain. Strain labels are shown in supplementary figure S3, Supplementary Material online.

Construction of phylogenies at four loci directly flanking the PaLoc integration site revealed different relationships between toxigenic clade 5 strains, nontoxigenic clade 5 strains, and representatives of other clades (fig. 5). The *cdu2*, *cdu1*, and *cdd2* genes in A^+^B^+^ and A^−^B^+^ clade 5 strains were more closely related to one another than with other clades, but gene *cdd1* showed a strikingly different pattern (fig. 5*C*). The same *cdd1* allele was present in all six A^+^B^+^ strains, as well as in the M120 reference strain and the closely related L033 strain, and this allele was more closely related to those found in clades 1–4, than in the A^−^B^+^ strains of clade 5 (fig. 5*C*). This suggests that *cdd1* may have been acquired along with the PaLoc in the A^+^B^+^ strains. This further supports the two independent PaLoc acquisitions by clade 5, possibly also involving homologous recombination of flanking sequences within the A^+^B^+^ strains. The deletion of most of the PaLoc in ST11(033) was estimated to be quite recent, occurring approximately 50 years ago.

### Clade 5 PaLoc Loss by Homologous Recombination

The phylogeny of the tree constructed using the core genomes of clade 5 isolates suggested that nontoxigenic strains have been generated by PaLoc loss from clade 5 genomes on multiple occasions, possibly by homologous recombination with nontoxigenic *C. difficile* strains (fig. 4). To investigate these losses in more detail, the genetic organization and phylogeny of loci flanking the PaLoc insertion site (Braun et al. 1996) in all available clade 5 genomes was compared with isolates representing the other four clades.

All toxigenic clade 5 strains (*n* = 18) and the atypical nontoxigenic WA12 contained a distinctive *cdu2* locus, lacking the 5’ terminal 600 bp of the gene and containing two frameshift mutations, a 228 bp deletion, and a 31 bp insertion with weak homology to *C. difficile* IStrons relative to CD630 (Sebaihia et al. 2006). A deletion coupled with an insertion also occurred in the *cdu3−cdu2* intergenic region (fig. 1*A, B*, and *E*). In contrast, the remaining nontoxigenic clade 5 isolates contained an intact *cdu2* gene, with the single exception of L033, due to its upstream deletion (fig. 1*D*). A neighbor-joining tree was built for *cdu2* using the 396 bp of the gene common to all isolates (fig. 5*A*). Although the number of polymorphic sites was low (65 variant sites), the *cdu2* fragment of toxigenic isolates was clearly distinct from all the nontoxigenic isolates except WA12 which clustered with the toxigenic-like clade 5. The remaining nontoxigenic clade 5 *cdu2* fragment clustered with the other four clades, away from toxigenic clade 5. Gene *cdu1* showed a very similar phylogenetic pattern to *cdu2* **(**fig. 5*B*). These data suggested that nontoxigenic clade 5 strains were generated (and whole *cdu2* genes acquired) during an interclade homologous recombination originating from nontoxigenic members of other clades.

**Fig. 6.—:**
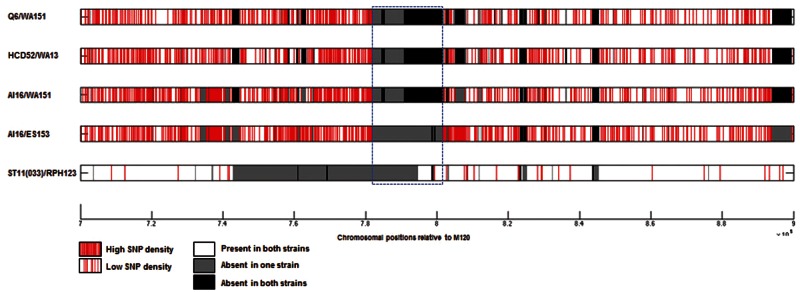
Investigation of mechanisms of PaLoc acquisition and modification post acquisition using SNP plots. The distribution of polymorphisms between five different pairs of strains is shown for the genomic region containing the PaLoc relative to the reference strain M120. The PaLoc region is indicated by the blue dashed box. Each row represents a pairwise comparison, and individual polymorphisms are shown in red.

To investigate whether any of the PaLoc losses had occurred recently, four pairs of closely related genomes were chosen from the whole-genome tree (fig. 4), with one nontoxigenic and one toxigenic strain within each pair. The lack of any clear increase or decrease in single nucleotide polymorphism (SNP) density between these pairs prevented the identification of recombination boundaries, probably because the PaLoc losses in these clade 5 isolates were not recent (fig. 6).

### Changes in the A^−^B^+^ Lineage PaLoc Postacquisition

In all of the isolates belonging to the clade 5 A^−^B^+^ lineage, there is an ORF at the 3′-end of the PaLoc (fig. 1*B*) closely related (79% identity at the amino acid level) to that seen at the PaLoc integration site of WA 12 (fig. 1*E*) This suggests that a mobile element may have been involved in the loss of *tcdA* and *tcdC* after the PaLoc was acquired.

Investigation of the phylogeny of the PaLoc *tcdE* gene across the *C. difficile* population structure revealed that the putative holin gene present in the A^−^B^+^ clade 5 PaLoc was only distantly related to that found in clades 1–4 (65–66% at the nucleotide level). It had slightly greater identity to *tcsE* from *C. sordellii* than the M120 *tcdE* (75% at the amino acid level vs. 71%) (fig. 5). Although BLAST searches of viral and bacterial databases were not able to identify a more closely related sequence, the A^−^B^+^ clade 5 *tcdE* was probably gained via allelic exchange from an unknown source.

## Discussion

Phylogenetic analysis of *C. difficile* clade 5 revealed its genetic diversity and highlighted the complex evolutionary history of its PaLoc. Clade 5 contained at least eight STs in addition to the well-characterized “hypervirulent” ST11. Two distinct phylogenetic branches were identified, suggesting the clade itself is not recently emergent as a clinical problem, in contrast to the emergent ST11(078) clone (Goorhuis et al. 2008; Walker et al. 2013). *Clostridium difficile* is estimated to have diverged from *Clostridium tetani* between 1.1 and 85 Ma, (He et al. 2010), although these two species are thought not to belong to the same family, let alone genus (Collins et al. 1994; Ludwig et al. 2009), making it difficult to date the origin of *C. difficile* as a species. Nevertheless, clade 5 is known to have diverged from the rest of the species very early on, making it potentially several million years old (He et al. 2010).

Fine-scale phylogenetic analysis suggested that clade 5 has acquired the PaLoc on two occasions. Clade 5 is relatively unusual in this respect, since clade 2 is the only other lineage in which multiple PaLoc variants have been identified to date (Dingle et al. 2011).

The exact mechanisms involved in PaLoc acquisition remain unclear, although the presence of an incongruent *cdd1* in the clade 5 A^+^B^+^ lineage implicates homologous recombination involving PaLoc flanking sequences in this case. An alternative mechanism that has been proposed is PaLoc acquisition by site-specific recombination, catalysed by an integrase supplied in trans (Dingle et al. 2014). This would be consistent with the single PaLoc integration site observed in clades 1–5, and the congruence of PaLoc flanking genes with clade (Dingle et al. 2014). Horizontal transfer of the PaLoc from strain CD630 to a nontoxigenic strain and consequent toxigenic conversion has been demonstrated (Brouwer et al. 2013).The PaLoc was transferred on variably sized DNA fragments, at a frequency similar to conjugative transposons seen in *C. difficile* (Brouwer et al. 2013). The first *C. difficile* genome to be sequenced contained an estimated approximately 10% mobile elements, hence there are a large number of candidates which could act as a helper element (Sebaihia et al. 2006).

It has been proposed that the ancestral *C. difficile* population may have been nontoxigenic, and that different PaLoc variants were acquired independently by five different clades after they diverged (Dingle et al. 2014). This was supported by the distribution of nontoxigenic strains throughout clades 1, 4 (Dingle et al. 2014), and 5, the distinct phylogeny of the PaLoc, and the existence of a divergent clade designated C-I, members of which have all been found to be nontoxigenic to date. Among the clade 5 isolates available to the present study, little evidence was found of a putative nontoxigenic ancestor, with analysis of *cdu2* suggesting all but one (WA12) of the nontoxigenic genotypes within this clade were originally toxigenic, but have lost their PaLoc via interclade homologous recombination with nontoxigenic isolates. The only nontoxigenic genotype to still have the signature clade 5 *cdu2*, isolate WA12, has lost part of its 115 bp PaLoc integration site upon insertion of the C-I clade-like sequence. SNP plots and fine-scale phylogeny suggested that none of these events was recent.

Previously reported data have shown that intraclade homologous recombination can involve sequences up to 234 kb (Dingle et al. 2014). Although the PaLoc appears to be stably integrated, like other regions of the *C. difficile* chromosome, it is susceptible to the activity of mobile elements, often resulting in large-scale deletions. The PaLoc seen in the toxinotype XXX/XXXI branch of clade 5 (fig. 1*B*) has a particularly complex history, with evidence of homologous recombination with other clades and potentially other species. A remnant of the C-I sequence may have been acquired during the same event which caused the large deletion at the 3′-end of the PaLoc. Fragments of putative mobile elements have been identified in the region of the deletion in the toxinotype X (A^−^B^+^) isolate 8864 (Song et al. 1999). The most common A^−^B^+^ type, UK 017, toxinotype VIII, which belongs to clade 4 has a approximately 1,200 bp deletion at the 3′-end of the *tcdA* gene which appears to have resulted from homologous recombination of the repeats within the cell-binding domain, in addition to a nonsense mutation further upstream (von Eichel-Streiber et al. 1999).

**Fig. 7.—:**
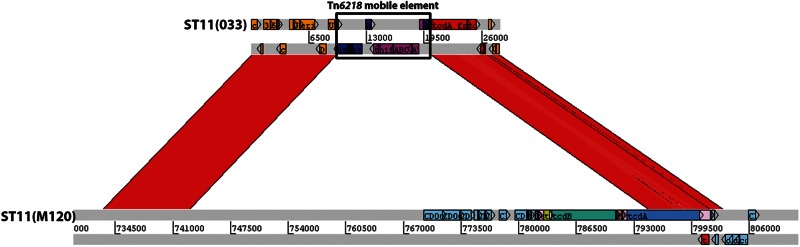
Modification of clade 5 PaLoc postacquisition by large-scale deletions. A comparison between the PaLoc regions of strains L033 (top) and M120 (bottom). The insertion of Tn*6218* (indicated in black box) has resulted in a deletion relative to M120 of 51,379 bp comprising most of the PaLoc and a large region upstream. Only *tcdC* and the 3′-terminal 2,456 bp of *tcdA* remain of the PaLoc in L033.

**Fig. 8.—:**
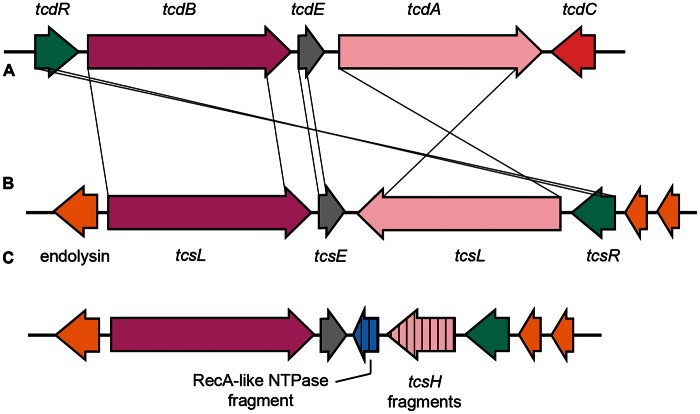
Genetic organization of the toxin-encoding loci of *C. difficile* and *C. sordellii*. The typical *C. difficile* PaLoc genetic organization (*A*) compared with the organization in *C. sordellii* strains VPI 9048 (*B*) and the toxin variant (tcsH^−^tcsL^+^) ATCC 9714 (*C*).

The most obvious evidence linking a partial deletion of the PaLoc to a mobile element occurs in L033, where Tn*6218* is present at the deletion site (fig. 7). Such deletions adjacent to mobile elements may be linked to failed attempts at transposition (Roberts et al. 1991). Also within the toxinotype XXX/XXXI lineage is a putative holin that appears to derive from an unknown source. The toxinotype XXX/XXXI PaLoc therefore appears to be the product of multiple evolutionary events including interspecies homologous recombination, and a possible insertion/deletion event involving a mobile element. However, not all mobile elements associated with the PaLoc have resulted in major deletions to it sequence. Tn*6218* is found within the clade 3 PaLoc near *tcdE* (Dingle et al. 2014) without the large deletions seen in the toxinotype XI PaLoc. A novel type of element, termed an IStron, has been found within *tcdA* (Braun et al. 2000). The *C. sordellii* PaLoc also seems to be susceptible to changes due to the activity of mobile elements. The fragment of an ORF near the large deletion of *tcsH* in *C. sordellii* ATCC 9714 (fig. 8) suggests that mobile elements are responsible for the generation of variant phenotypes (TcsH^−^TcsL^+^) in this species as well. It was hoped that the availability of the toxin-encoding locus of the reference *C. sordellii* isolate would further enhance our understanding of the *C. difficile* PaLoc. The *C. sordellii* toxins are more closely related to those of *C. difficile* than any of the other LCT (large clostridial toxin) family, and the presence of two homologs accompanied by a putative holin hinted at a similar arrangement (fig. 8). However, data available to this study indicate that the *C. sordellii* toxin locus is apparently part of a much larger DNA element (possibly a plasmid), but its borders could not be defined (Elliott B, Hong S, Knight DR, Riley TV, unpublished data). The genome sequences from *C. sordellii* ATCC 9714 and VPI 9018 (TcsH^+^TcsL^+^) have been published, and the authors were also unable to properly define the element (Sirigi Reddy et al. 2013). The origins of Pathogenicity Islands are particularly enigmatic in gram-positive organisms (Hacker et al. 2004). The presence of a putative holin and lysin within the PaLoc is suggestive of a bacteriophage origin, and alpha toxin (TcnA) of *C. novyi* is still carried by a bacteriophage (Eklund et al. 1976).

*Clostridium difficile* clade 5 exhibits significantly more genetic diversity than previously acknowledged. The level of diversity among Australian clade 5 isolates is intriguing. Certain clades are associated with particular geographical areas (e.g., clade 4 with South East Asia, clade 2 with North America). It is possible that clade 5 may have originated in Australia; however, further investigation is required.

The two main lineages within clade 5 appear to have acquired the PaLoc independently, but possibly from a similar source. The loss of the PaLoc via interclade homologous recombination has occurred on several occasions, resulting in nearly all the nontoxigenic strains in this study. A number of evolutionary events resulting in significant changes to the clade 5 PaLoc were identified in this study, showing that the PaLoc is continually evolving. In order to better understand *C. difficile* and its evolution as a pathogen, we must continue to study its phylogeny and the evolutionary history of the PaLoc.

## Supplementary Material

Supplementary figures S1–S3 are available at *Genome Biology and Evolution* online (http://www.gbe.oxfordjournals.org/).

Supplementary Data
