# Supplementary material for: The Complexity and Diversity of the Pathogenicity Locus in Clostridium difficile Clade 5
Source: Genome Biol Evol. 2014 Dec 10;6(12):3159–70. doi: 10.1093/gbe/evu248 (PMC4986448; doi:10.1093/gbe/evu248)
Supplement: Supplementary Data [file supp_6_12_3159__index.html]

The complexity and diversity of the Pathogenicity locus in Clostridium difficile clade 5 — The Complexity and Diversity of the Pathogenicity Locus in Clostridium difficile Clade 5 — Supplementary Data 

# The Complexity and Diversity of the Pathogenicity Locus in *Clostridium difficile* Clade 5

## Supplementary Data

files

**Files in this Data Supplement:**

- Supplementary Data - tif file
- Supplementary Data - tif file
- Supplementary Data - tif file
