# Supplementary figures and images for: The Complexity and Diversity of the Pathogenicity Locus in Clostridium difficile Clade 5
Source: Genome Biol Evol. 2014 Dec 10;6(12):3159–70. doi: 10.1093/gbe/evu248 (PMC4986448; doi:10.1093/gbe/evu248)

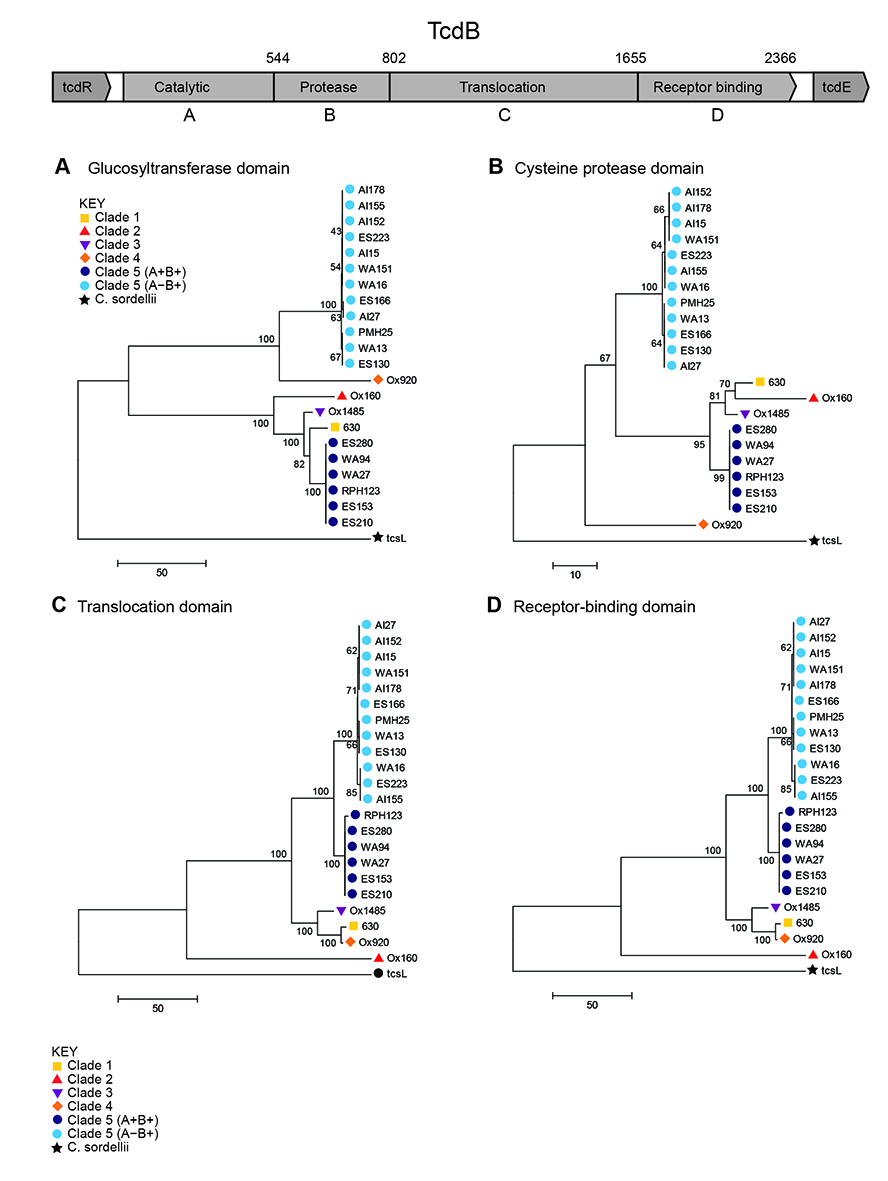

Supplement: Supplementary Data [file supp_evu248_suppl_figure_1.tif]

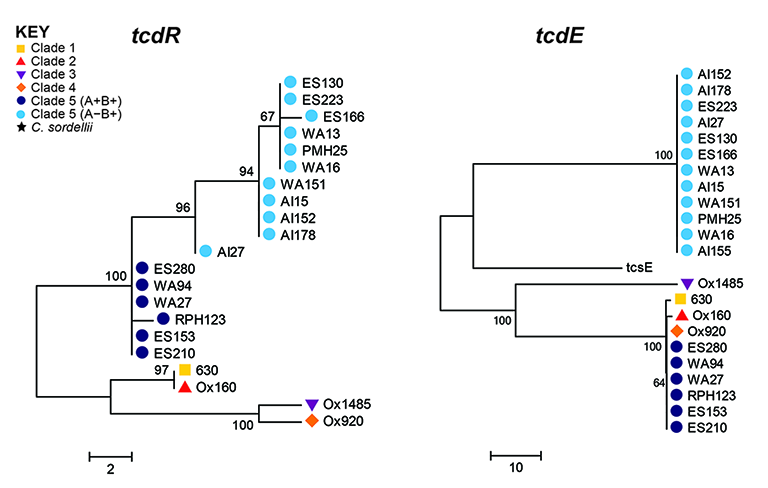

Supplement: Supplementary Data [file supp_evu248_suppl_figure_2.tif]

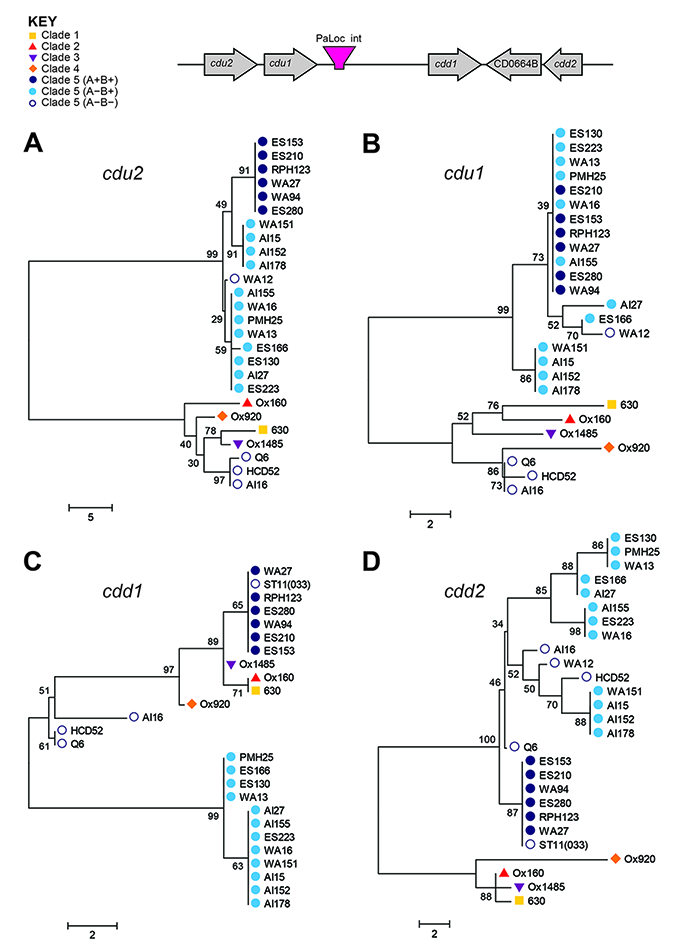

Supplement: Supplementary Data [file supp_evu248_suppl_figure_3.tif]
